# AKE - the Accelerated *k*-mer Exploration web-tool for rapid taxonomic classification and visualization

**DOI:** 10.1186/s12859-014-0384-0

**Published:** 2014-12-13

**Authors:** Daniel Langenkämper, Alexander Goesmann, Tim Wilhelm Nattkemper

**Affiliations:** Biodata Mining, Bielefeld University, Universitätsstraße 15, Bielefeld, Germany; Bioinformatik und Systembiologie, Justus Liebig University, Düsternbrooker Weg 20, Gießen, Germany

**Keywords:** Metagenomics, Classification, Acceleration, Web-based, H2SOM, k-mer

## Abstract

**Background:**

With the advent of low cost, fast sequencing technologies metagenomic analyses are made possible. The large data volumes gathered by these techniques and the unpredictable diversity captured in them are still, however, a challenge for computational biology.

**Results:**

In this paper we address the problem of rapid taxonomic assignment with small and adaptive data models (< 5 MB) and present the accelerated *k*-mer explorer (AKE). Acceleration in AKE’s taxonomic assignments is achieved by a special machine learning architecture, which is well suited to model data collections that are intrinsically hierarchical. We report classification accuracy reasonably well for ranks down to order, observed on a study on real world data (Acid Mine Drainage, Cow Rumen).

**Conclusion:**

We show that the execution time of this approach is orders of magnitude shorter than competitive approaches and that accuracy is comparable. The tool is presented to the public as a web application (url: https://ani.cebitec.uni-bielefeld.de/ake/, username: bmc, password: bmcbioinfo).

**Electronic supplementary material:**

The online version of this article (doi:10.1186/s12859-014-0384-0) contains supplementary material, which is available to authorized users.

## Background

Metagenomics is the direct sequencing and analysis of environmental samples. Metagenomic studies are used in a variety of fields including, e.g. bio-medical studies [1] and ecological diversity studies [2]. As a first step after sequencing taxonomic composition is estimated and taxonomic categories are assigned to the data. This is a challenging problem due to sequence length and complexity of the data captured [2]. For analysis of the taxonomic composition the analysis of 16S rRNA sequences is a prominent step, see [3,4]. This imposed some limitations, e.g. the copy number can vary by an order of magnitude [5] and therefore we will focus on whole metagenome analysis. Multiple tools exist that are able to predict the class of a genomic sample sequence, most of them using alignments (e.g. Megan4 [6], (Web)Carma3 [7,8], MG-Rast [9]). As these can be very time consuming, alternative approaches based on profile features have been proposed (Phylopythia(S) [10,11], NBC [12], TAC-ELM [13], TAXSOM [14], PhymmBL [15], Kraken [16], taxy [17]). Sequence data are transformed to profile features, i.e. feature vectors that consist of various measurements describing the nucleic composition of the sequence. Frequently employed characteristics are G/C content [18] and *k*-mer occurrence [19,20]. The speedup of these techniques is traded in for a loss of accuracy, compared to the alignment-based methods. Nevertheless, it has been shown that *k*-mer profiles are distinctive enough for binning in metagenomic studies and for classification up to certain levels in the tree of life [21]. For benchmarking we compared AKE with Phylopythia(S) and NBC. Phylopythia classifies profile features with a SVM-based classifier architecture. The web-based version is called PhylopythiaS. It uses two different models, either a generic model for classification or a sample specific one, which can be generated by the user prior to classification. We benchmarked against the parameterless generic model. NBC implements the naive Bayes classifier for taxonomic assignment as a web application. The *k*-mer length as well as the genomes to match against can be chosen. We chose the Bacteria/Archea genomes to match against and a *k*-mer length of 6 for benchmarking.

This paper presents AKE (Accelerated *k*-mer Exploration web-tool) a computational approach to rapid taxonomic assignment for an immediate response to new data. A rapid taxonomic assignment can be of interest, when data sets from lots of samples are to be analyzed immediately or new data sets are generated rapidly by filtering and fusion. A result of AKE is a rapid taxonomic assignment presented as a web-based, interactive and dynamic visualization. AKEs computational speed is achieved by (1) using refined *k*-mer profile features [21], (2) a data-driven, i.e. learned, hierarchical and descriptive model, which provides the basis for classification and visualization, and (3) parallel computing. This work is based on a previous paper by Martin et al. [21] sharing the features and binning method, namely the H ^2^SOM. However, the classifier architecture is different and Martin et al. do not provide a web interface for visualization of results. Furthermore, the execution speed is increased by using parallelization and a faster implementation. To boost classification accuracy a rejection class is introduced to the model containing non-specific profiles. This results in a web accessible system for low performance computers that features an immediate first visual inspection of new data, i.e. some data might be rejected if it is unspecific. The accuracy is comparable to similar approaches but with a faster execution time. The tool is publicly available as a web application (https://ani.cebitec.uni-bielefeld.de/ake/, username: bmc, pw: bmcbioinfo), which facilitates the ease of use. This releases the users of the burden of resourceful operations on their own systems, e.g. analyses on small-scale computers in laboratories are made possible. Furthermore, no software packages have to be downloaded and installed. The only requirement is an up-to-date web-browser (≈ not older than 2 years).

Recent reports of IMG4 [[22], progress report (http://img.jgi.doe.gov/w/doc/releaseNotes.pdf)] show a rapidly growing amount of available metagenomes. Likewise, the PubMed hits for the term “metagenomics” grew massively in the latter years, showing the importance of the field.

The following Methods section describes the features, methods and data used in this study and how these are used to build a classification system for metagenomic data. In the Results and discussion section we present the performance on two real world data sets, compared to similar approaches. Furthermore, the differences in runtime are reported. The Conclusion sums up the results of this study.

## Methods

**Figure 1:**
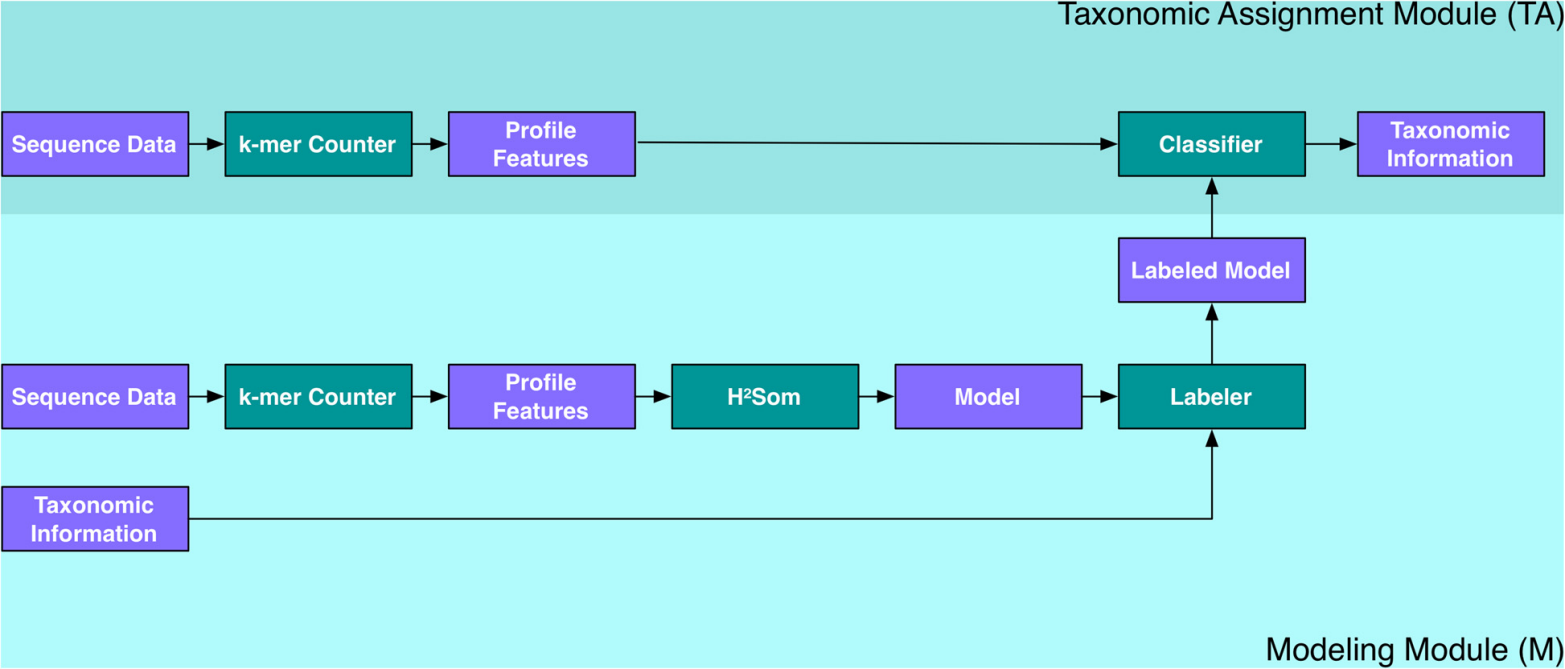
**Workflow of AKE.** Blue boxes are data, whereas green ones are applications.

As can be seen in Figure 1 AKE consists of two modules: taxonomic assignment (TA) and modeling (M). In the M-module, a reference set of genome sequences *Γ*_ref_={*S*^(*ζ*)^ } is used to learn a model that describes the function for assignment of taxonomic classes to sequence reads *S*^(*ζ*)^ based on a read’s profile feature **x**^(*ζ*)^. For assigning new sequence data *Γ*_new_{*S*^(*ξ*)^} with the TA-module, these reads are also represented by profile features **x**^(*ξ*)^ and those are assigned to taxonomic classes. The composition of all assignments of *Γ*_new_{*S*^(*ξ*)^} are visualized in a dynamic and interactive web-tool.

### *k*-mer features

For using the sequences *S*^(*ζ*)^*ζ*=0,…,*n* with a mathematical model like the H ^2^SOM, features **x**^(*ζ*)^*ζ*=0,…,*n* have to be computed for the sequence reads. For this purpose *k*-mer profiles with three different normalizations are used and referred to as $\left [\textbf {x}^{(\zeta)}_{\text {tf}},\textbf {x}^{(\zeta)}_{\text {tfti}},\textbf {x}^{(\zeta)}_{\text {oligo}}\right ]$. They are listed here with basic explanations, further information can be found in [21].

A *k*-mer *κ*_*j*_(*k*,*Σ*) is a word of length *k* on an alphabet *Σ*. In this case *Σ*={*a*,*c*,*g*,*t*} is the DNA alphabet and therefore 4^*k*^*k*-mers $\phantom {\dot {i}\!}\kappa _{j (j=0,\ldots,4^{k}-1)}(k,\Sigma)$ exist. Let $\phantom {\dot {i}\!}t_{j}^{(\zeta)}$ be the number of occurrences of the *k*-mer *κ*_*j*_(*k*,*Σ*) in sequence *S*^(*ζ*)^, $\mathcal {C}_{\kappa }(\kappa _{j}(k,\Sigma)$ a function counting these occurrences and *S*^′^ a substring of *S* matching the specified *k*-mer.

(1) $$ \begin{aligned} t_{j}^{(\zeta)}&=\mathcal{C}_{\kappa}\left(\kappa_{j}(k,\Sigma),S^{(\zeta)}\right)\;\,\text{with}\\ \mathcal{C}_{\kappa}\left(\kappa_{j}(k,\Sigma),S^{(\zeta)}\right) &=\left\lvert \left\{S^{\prime} \in S^{(\zeta)} \lvert S^{\prime} =\kappa_{j}(k,\Sigma)\right\}\right\rvert \end{aligned}  $$

A *k*-mer-profile $K^{(\zeta)}(k,\Sigma)\in \mathbb {N}^{4^{k}}$ is defined as

(2) $$ K^{(\zeta)}(k,\Sigma)=\left(t_{0}^{(\zeta)},t_{1}^{(\zeta)},\ldots,t_{4^{k}-1}^{(\zeta)}\right)  $$

For the sake of compactness we omit the term (*k*,*Σ*) for *K*^(*ζ*)^(*k*,*Σ*) and *κ*_*j*_(*k*,*Σ*) in the following text. The term frequency features (tf) are gained by normalizing every *k*-mer profile to unit length.

(3) $$ \textbf{x}^{(\zeta)}_{\text{tf}}=\frac{K^{(\zeta)}}{\left\Vert K^{(\zeta)} \right\Vert}  $$

By taking into account the abundance of a certain *k*-mer in all *k*-mer-profiles we gain the term frequency term importance (tfti) weighted features. Let $t_{j}=\sum \limits _{\zeta } t_{j}^{(\zeta)}$ denote the sum of frequencies of *k*-mer *κ*_*j*_ in all *k*-mer-profiles in *Γ*_ref_. Let $t^{(\zeta)}=\sum \limits _{0}^{4^{k}-1} t_{j}^{(\zeta)}$ be the sum of all frequencies for a sequence *S*^(*ζ*)^. Therefore, we compute the tfti-weighted features for every *k*-mer profile as:

(4) $$ \textbf{x}^{(\zeta)}_{\text{tfti}}=\left(\frac{t_{0}^{(\zeta)}}{t_{0} t^{(\zeta)}},\frac{t_{1}^{(\zeta)}}{t_{1} t^{(\zeta)}},\ldots,\frac{t_{4^{k}-1}^{(\zeta)}}{t_{4^{k}-1} t^{(\zeta)}}\right)  $$

To reduce a bias towards frequent *k*-mers the vectors are normalized to unit length.

Considering the over- and under-representation of *k*-mers in one sequence compared to the others we compute the oligo features (oligo). Therefore, the occurrence of each *k*-mer is computed and the expected occurrence of it is estimated. Let

$$\begin{aligned} p^{(\zeta)}(\eta)&=\frac{1}{\lvert S^{(\zeta)} \rvert}\mathcal{C}_{\Sigma}(\eta)\ \text{with}\ \eta \in\Sigma,\\ \mathcal{C}_{\Sigma}\!\left(\eta,S^{(\zeta)}\right)&=\left\lvert \left\{\eta^{\prime} \in S^{(\zeta)} \lvert \eta^{\prime}=\eta\right\}\right\rvert \end{aligned} $$

be the probability to observe a certain nucleotide *η* in a sequence *S*^(*ζ*)^ with a sequence length |*S*^(*ζ*)^| and let *η*^′^ be a nucleotide in the sequence *S*^(*ζ*)^ matching a specified nucleotide. Let $E^{(\zeta)}(\kappa _{j})\approx \lvert S^{(\zeta)}\rvert \prod \limits _{l=0}^{k-1} p^{(\zeta)}(\kappa _{j,l})$ (with *κ*_*j*,*l*_ referring to the l-th symbol in *κ*_*j*_) be an estimate for the occurrence of a *k*-mer *κ*_*j*_ in a sequence *S*^(*ζ*)^. The contrast of expectation and observation is

$$g^{(\zeta)}(\kappa_{j})=\left\{\begin{array}{cl} 0, & \text{if}\,\, K^{(\zeta)}_{j} = 0 \\ \frac{K_{j}^{(\zeta)}}{E^{(\zeta)}(\kappa_{j})}, & \text{if}\,\, K_{j}^{(\zeta)}>E^{(\zeta)}(\kappa_{j})\\-\frac{E^{(\zeta)}(\kappa_{j})}{K_{j}^{(\zeta)}}, & \text{else} \end{array}\right. $$

The oligo features are computed for each *k*-mer as

(5) $$ \textbf{x}^{(\zeta)}_{\text{oligo}}=\left(g^{(\zeta)}(\kappa_{0}),g^{(\zeta)}(\kappa_{1}),\ldots,g^{(\zeta)}\left(\kappa_{4^{k}-1}\right)\right)  $$

### The H2SOM classifier

For creating a descriptive model of the *k*-mers a Hyperbolic Self Organizing Map is used. The Self Organizing Map is a neural network proposed by Teuvo Kohonen [23]. Many variants have been proposed since, but all share the basic setup that consists of a set of neurons (**u**_*i*_,*z*_*i*_)_*i*=1…*I*_ that are arranged in a grid with *z*_*i*_ being the grid coordinate and **u**_*i*_ being the attached neural unit also called the prototype. The architecture of the grid differs by the type applied.

In the Hyperbolic SOM (HSOM) [24] the algorithm is defined in non-euclidean space. The Hyperbolic Hierarchical SOM (H ^2^SOM) [25] as used in this paper introduces a hierarchical grid structure to the hyperbolic version.

In metagenomics, the H ^2^SOM has been applied already for visual exploration and binning [21]. In [26] it was shown, that clustering genome data with a HSOM correlates more to the tree-of-life structure than the standard SOM clustering.

**Figure 2:**
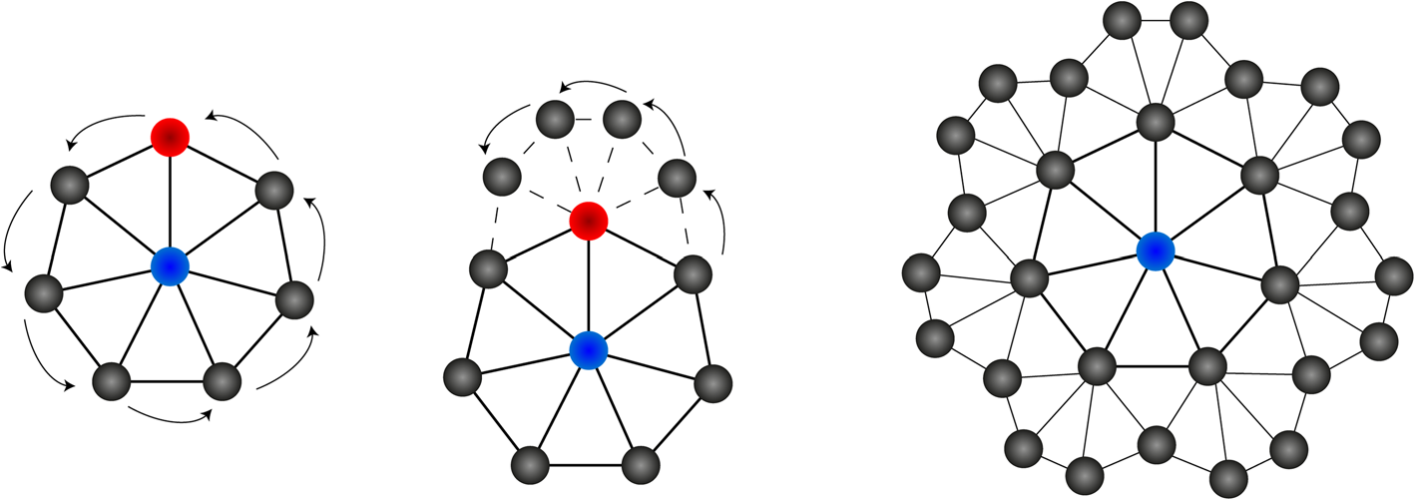
**Architecture of H** ^**2**^ **SOM.** The construction of a H ^2^SOM grid with *s*=7 and *r*=2. A Möbius transform is used in a recursive way to create a regular shaped hierarchical grid in hyperbolic space.

The network is built by placing a central neuron and spawning its *s*−1 children around it using the Möbius transformation. This is done recursively for every neural unit until all have *s* neighbors and the maximum number of rings *r* is reached. Hereby *s*−3 neighbors are placed as children, 2 as siblings and 1 already exists as parent. For further information refer to [25] or see Additional file 1. An example of a H ^2^SOM grid with two rings and seven neighbors (*s*=7,*r*=2) is shown in Figure 2.

The learning of a non-euclidean SOM is done equivalently to an euclidean SOM using a reference set *Γ*_ref_={**x**^(*ζ*)^}, but with a refined neighborhood function (Eq. 6) taking the change from euclidean to hyperbolic space into account.

(6) $$\begin{array}{@{}rcl@{}}  h(i,i^{\prime}) = \exp{\left(- \frac{\arctan{\left(\left| \frac{z_{i}-z_{i^{\prime}}}{1 - \bar z_{i} z_{i^{\prime}}} \right| \right)}}{\sigma^{2}(t)}\right)}. \end{array} $$

The number of neural units in the grid of a H ^2^SOM grows exponentially with the number of rings *r*. This leads to a more trustworthy mapping but dramatically increases the time required for the search for the best matching unit (BMU) during training. Leveraging the hierarchical structure of the grid a beam search is applied to approximate the global BMU in each training step. The search starts with the central neural unit as the initial BMU. For a beam width of *w*=1 it continues by recursively choosing the BMU among the children of the last winning neural unit until it reaches the current periphery of the grid. The BMU determined for the last ring is an approximation of the global BMU. For values of *w*>1 searching is done equivalently, but the children of *w* different neural units are searched for the BMU. It has been shown in [25] that this strategy accelerates the training significantly while staying close to or even surpassing the performance using global search.

The H ^2^SOM depends on parameters that need to be optimized. These are the number of rings *r*, the spread factor *s*, the neighborhood adaption modifier *n* and the learning rate *ε*. The algorithm is very robust against changes in *ε* and *n* but the parameters determining size and architecture (*r*,*s*) are important. By employing cross validation the parameters *r*=5 and *s*=8 were determined to create a good descriptive model (see Additional file 2).

### Taxonomic labeling of unsupervised neural networks

After training the H ^2^SOM neural units are linked to semantics, i.e. taxonomic categories. To this end, the labeled training data *Γ*_ref_{(**x**^(*ζ*)^,*L*^(*ζ*)^)}, where *Γ*_ref_ is a set of features with their respective labels, are mapped to the H ^2^SOM. This is done with a labeling function $\mathcal {L}(\textbf {u}_{i})$ that is defined on the Voronoi cell *V*(**u**_*i*_) of the training data for each prototype

$${\small\begin{aligned} V(\textbf{u}_{i}) : V(\textbf{u}_{i})=\left\{\textbf{x}^{(\zeta)} \in \Gamma_{\text{ref}} \lvert d\!\left(\textbf{x}^{(\zeta)},\textbf{u}_{i}\right)<d\!\left(\textbf{x}^{(\zeta)},\textbf{u}_{j}\right), \forall i\neq j\right\} \end{aligned}} $$

using a given metric *d* (in our case the euclidean metric). We propose two approaches: majority voting $\mathcal {L}^{\text {maj}}$ and purity voting $\mathcal {L}^{\text {pur}}$ defined as

(7) $$ \begin{aligned} \mathcal{L}^{\text{maj}}(\textbf{u}_{i})&=\arg\max_{l} (\Psi (V(\textbf{u}_{i}),l))~\text{with}\\ \Psi (V(\textbf{u}_{i}),l) &= \left\lvert\left\{\textbf{x}^{(\zeta)} \in V(\textbf{u}_{i})\lvert L^{(\zeta)}=l\right\}\right\rvert \end{aligned}  $$

and

(8) $$ \mathcal{L}^{\text{pur}}(\textbf{u}_{i})=\left\{\begin{array}{cl} \mathcal{L}^{\text{maj}}(\textbf{u}_{i}), & \text{if}\,\, \Psi(V(\textbf{u}_{i}),\mathcal{L}^{\text{maj}}(\textbf{u}_{i}))> \alpha \\ \mathcal{R}, & \text{else} \end{array}\right.,  $$

with  being a special label namely the rejection class and *α* a threshold value.

### Classification rules

A H ^2^SOM labeled in one of the above ways can be used for classification. To assign a sample *ξ* (a sequence), the profile feature vector **x**^(*ξ*)^ is computed, employing the same *k*-mer normalization strategy as used for labeling the model. For assignment a particular function $\mathcal {C}\left (\textbf {x}^{(\xi)}\right)$ is chosen from $\left [\mathcal {C}^{\text {nn}}\left (\textbf {x}^{(\xi)}\right),\mathcal {C}^{\text {thresh}}\left (\textbf {x}^{(\xi)}\right), \mathcal {C}^{\text {nbrs}}\left (\textbf {x}^{(\xi)}\right)\right ]$, defined in the following.

The most straightforward function is to assign **x**^(*ξ*)^ to the label $\mathcal {L}({\textbf {u}_{j}})$, which is assigned to the nearest neighbor **u**_*j*_ in the model.

(9) $$ \mathcal{C}^{\text{nn}}\left(\textbf{x}^{\xi}\right) =\mathcal{L}({\textbf{u}_{j}})~\text{with}~j= \left(\arg\min_{i} d\left(\textbf{x}^{(\xi)},\textbf{u}_{i}\right)\right)  $$

Furthermore, the distance function *d*(**x**^(*ξ*)^,**u**_*j*_) can be seen as a certainty measure that the BMU **u**_*j*_ is the correct association of **x**^(*ξ*)^. Therefore, we define an arbitrary threshold *β* beyond which the association is assumed to be uncertain. The value of *β* is empirically determined.

(10) $$ {\small\begin{aligned} &\mathcal{C}^{\text{thresh}}\left(\textbf{x}^{(\xi)}\right)\\ &\;=\left\{ \!\begin{array}{cl} \mathcal{C}^{\text{nn}}\left(\textbf{x}^{(\xi)}\right), & \text{if} \,\,d\left(\textbf{x}^{(\xi)},\textbf{u}_{j}\right)< \beta~\text{with}~j=\arg\min\limits_{i} d\left(\textbf{x}^{(\xi)},\textbf{u}_{i}\right) \\ \mathcal{R}, & \text{else} \end{array}\right. \end{aligned}}  $$

The previous strategies determine the label in a Winner-Takes-All (WTA) manner. But the H ^2^SOM has the property that neighboring neural units, i.e. grid neighbors, share common properties, usually referred to as “neighborhood preservation”. The third version uses this feature to reduce the number of false positive classifications. To this end, the neighborhood of a BMU is evaluated to smooth out unlikely assignments with a large BMU distance and “taxonomic disagreement” to the neighborhood.

(11) $$ {\small\begin{aligned} &\mathcal{C}^{\text{nbrs}}\left(\textbf{x}^{\xi}\right)\\ &\;=\!\left\{\!\begin{array}{cl} \mathcal{L}({\textbf{u}_{j+1}}), & \text{if} \,\, d\!\left(\textbf{x}^{(\xi)\!},\textbf{u}_{j+1}\right)\,+\,d\!\left(\textbf{x}^{(\xi)}\!,\textbf{u}_{j-1}\right) \!<\! 3*d\!\left(\textbf{x}^{(\xi)}\!,\textbf{u}_{j}\right) \!\wedge \\ &\mathcal{L}({\textbf{u}_{j-1}\!})=\mathcal{L}({\textbf{u}_{j+1}\!})~\text{with}~j=\arg\min\limits_{i} d\!\left(\textbf{x}^{(\xi)\!},\textbf{u}_{i}\right) \\ \mathcal{C}^{\text{nn}}\left(\textbf{x}^{(\xi)}\right), & \text{else} \end{array}\right. \end{aligned}}  $$

For training sequences exceeding 4 kb from the NCBI full genome database (bacteria/virus, 2014/04/13) were used. A list of GI numbers (http://www.ncbi.nlm.nih.gov/Class/FieldGuide/glossary.html\#GI) is provided (see Additional file 3). Out of these sequence data four different data sets were generated for model building. Therefore, the sequences *S*^(*ζ*)^ were cut at different length $\left (\text {15 kb, 4 kb,}\; \frac {|S^{(\zeta)}|}{2}, \frac {|S^{(\zeta)}|}{4}\right)$.

## Results and discussion

For parameter optimization and model evaluation a cross validation study (see Additional file 2) was done. The most promising *k*-mer length was determined to be *k*=4. For larger values of *k* the classification accuracy increased partly, however a decrease in speed can be observed. The two labeling strategies (Eqs. 7, 8), for building the taxonomic model, combined with the three different classification algorithms (Eqs. 9, 10, 11) were applied. A trade-off between correctness of assignment and number of rejections was observed for all six variants. A good balance between assignment correctness, number of rejections and execution speed was determined using purity voting (Eq. 8) for model construction and nearest neighbor selection (Eq. 9) for taxonomic assignment.

Thus, for the following real world data set examples, purity voting with a threshold of *α*=0.8 for labeling (Eq. 8) and the nearest neighbor strategy (Eq. 9) for assignment were the most promising settings compared to the other variants. For the H ^2^SOM algorithm an architecture with *r*=5 rings and *s*=8 neighbors was chosen.

### Acid mine drainage

The Acid Mine Drainage data set [27] was taken at Iron Mountain in California. The community is comprised of five high abundant species namely Ferroplasma Types I and II, a Thermoplasmatales species, all of phylum Euryarchaeota, and Leptospirillum sp. Group I and II of phylum Nitrospirae. The data has been received from DOE Joint Genome Institute (http://img.jgi.doe.gov (taxon 2001200000)) along with its taxonomic affiliation and is build of 1183 scaffolds of approximately 10 Mb of sequence information.

We compared AKE with some similar approaches including NBC [12] and PhyloPythiaS [11] with generic and sample specific model. All results were obtained using a model derived from the 15 Kb data set of NCBI genomes mentioned above. We did not explore the possibility to generate a sample specific model as described in [11], but expect it to have a similar positive influence as in the cited study. When using the web service the parameters given above are applied.

**Figure 3:**
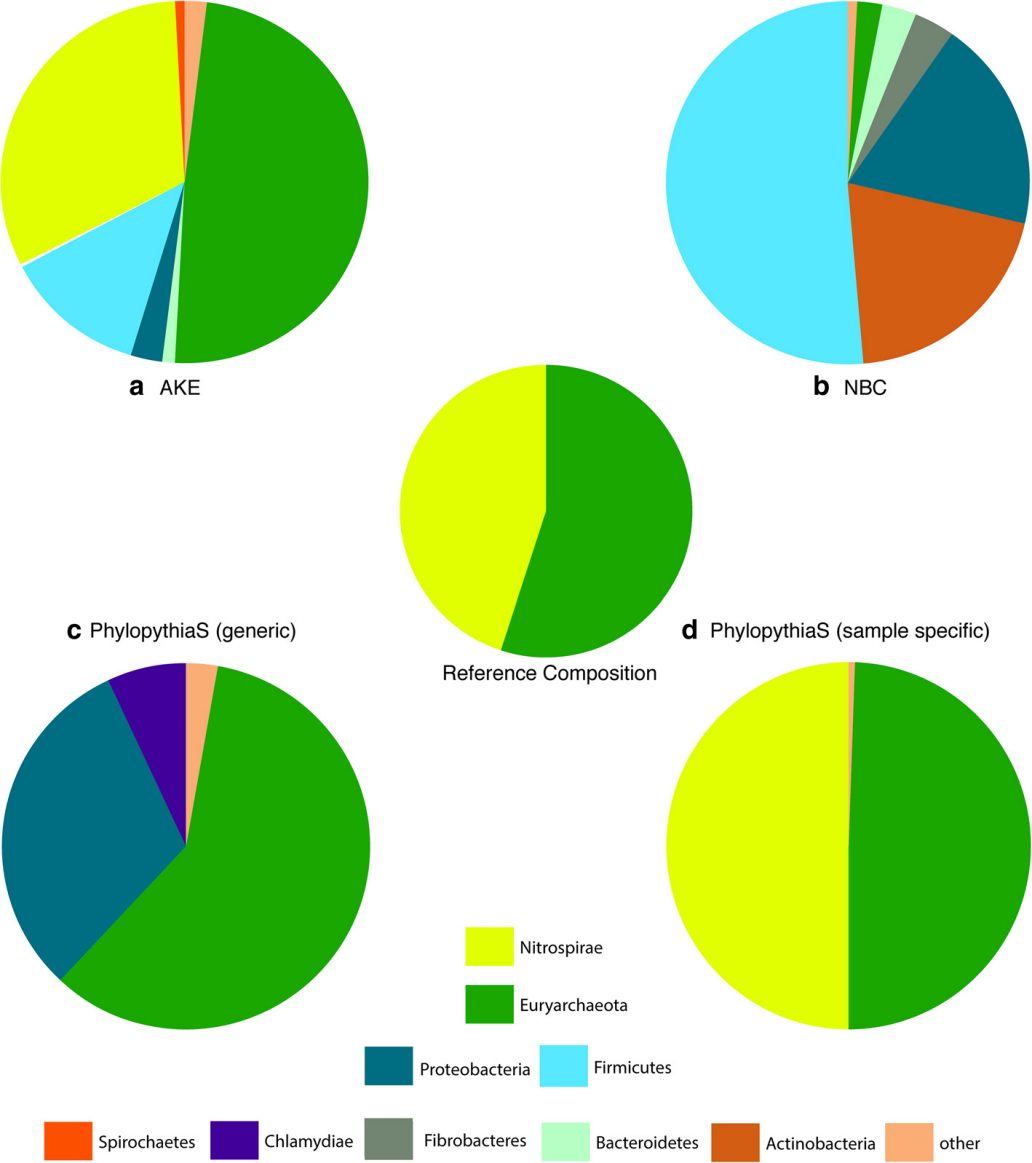
**Results of the AMD study.** Comparison of AKE/NBC/PhylopythiaS with a generic model/PhylopythiaS with a sample specific model. Data for NBC/PhylopythiaS derived from [11].

The high abundant species are Thermoplasmatales archaeon Gpl (410), Leptospirillum sp. Group II (70), Leptospirillum sp. Group III (474), Ferroplasma acidarmanus Type I (170), Ferroplasma acidarmanus Type II (59). When looking at the results (Figure 3) we see that AKE outperforms NBC and PhylopythiaS (generic model). But it is outperformed by PhylopythiaS employing a sample specific model.

### Cow rumen

**Figure 4:**
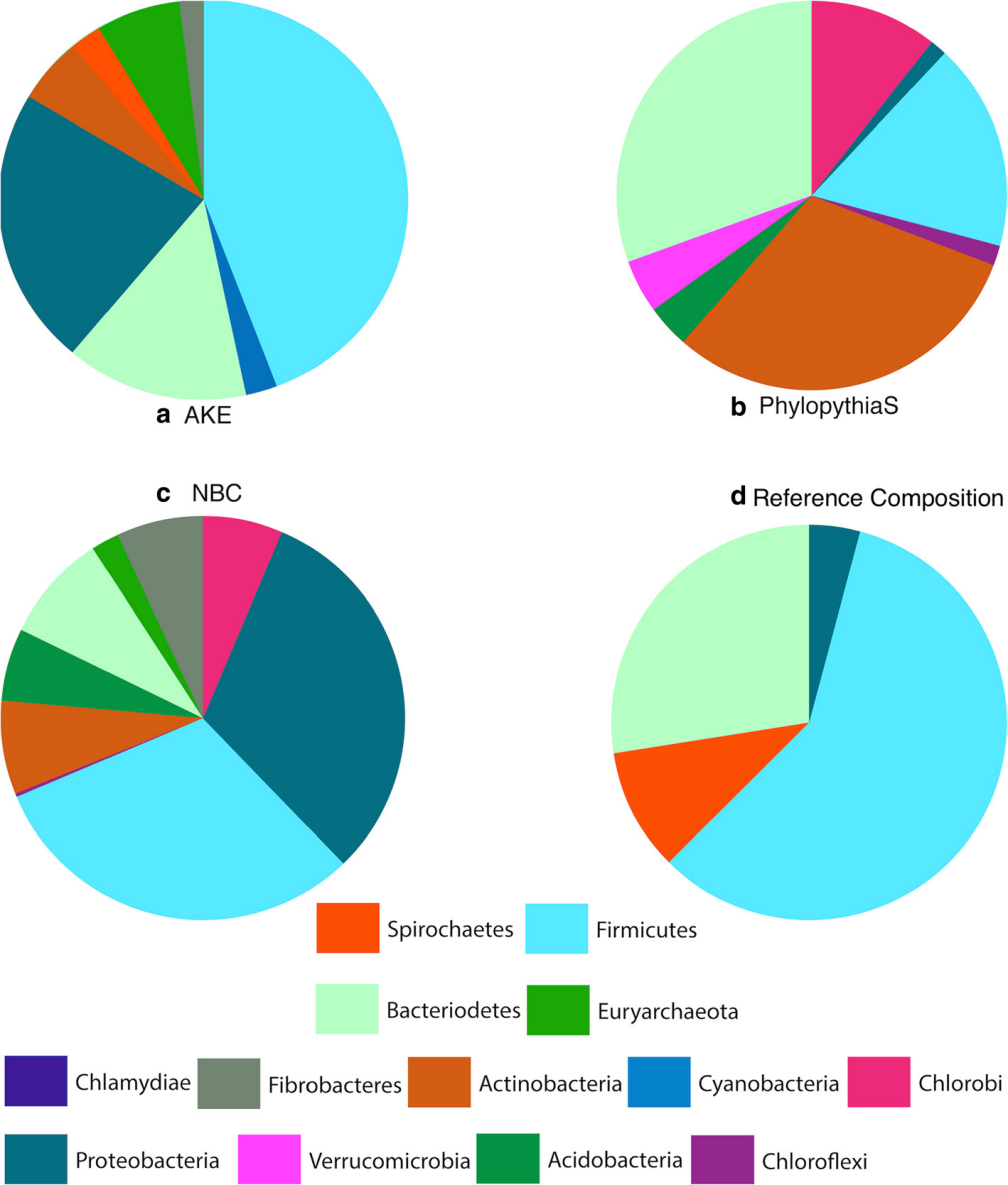
**Results of cow rumen bins study.** Comparison of AKE/NBC/PhylopythiaS with a generic model.

The Cow Rumen data set consists of a community taken from the deconstruction process of switchgrass in a cow rumen [28]. The cited study could identify 15 draft genomes with completeness between 60% and 93%. On the phylogenetic level of order these samples are comprised of Spirochaetales, Clostridiales, Bacteroidales and Myxococcales. Since a gold standard for all scaffolds does not exist, this reference composition (see Figure 4d)) has to be taken as a rough estimate. The data has been received from NERSC Science Gateways (http://portal.nersc.gov/project/jgimg/CowRumenRawData/submission/). An assignment for the genomic bins (cow_rumen_genome_bins.tar.gz) as well as for the scaffolds (cow_rumen_fragmented_velvet_assembly_scaffolds.fas.gz) is provided. We compared PhylopythiaS (generic model) and NBC with AKE. When looking at the results (Figure 4) we see that AKE outperforms NBC and predicts slightly better than PhylopythiaS.

### Online resources

Please note that further classification results are provided online within AKE. These include the results of the AMD and cow rumen data sets with classification down to order as well as a reference composition for these data sets visualized with AKE. Furthermore, the analysis of simulated data sets [29] is provided.

### Execution times

The application is written in Python using a C extension for fast computation. The authors implemented the *k*-mer counting as well as the H ^2^SOM. The execution times are measured using Python’s *time()* function. All experiments were repeated ten times and the mean value of this is stated below. The machine used, is the same web server that serves the results for the web interface. It is a virtual machine running two Intel Xeon E5450 CPUs at 3 GHz with 32 GB main memory operated by Sun Solaris 10. The application is multi-threaded using 4 threads.

The execution times are dominated by the counting of *k*-mers, which is heavily influenced by I/O load on the system (see Table 1). For faster loading all data resides on a tmpfs filesystem (a RAMdisk like filesystem). It is to note that the times were measured with a standalone non-CGI application. A little overhead using CGI can be expected as well as some time for uploading of data.

**Table 1:** **Execution times of AKE**

**Data set**	**#Sequences**	**Megabases**	**Runtime**	**Runtime**
			**(** ***k*** **-mers)**	**(Assignment)**
			**in s**	**in s**
AMD	1183	10.83	0.63	0.43
Cow rumen (bins)	466	34.14	0.71	0.21
Cow rumen (scaffolds)	26042	568.59	13.68	10.4

Measures for typical metagenome data sets.

### The web-application

**Figure 5:**
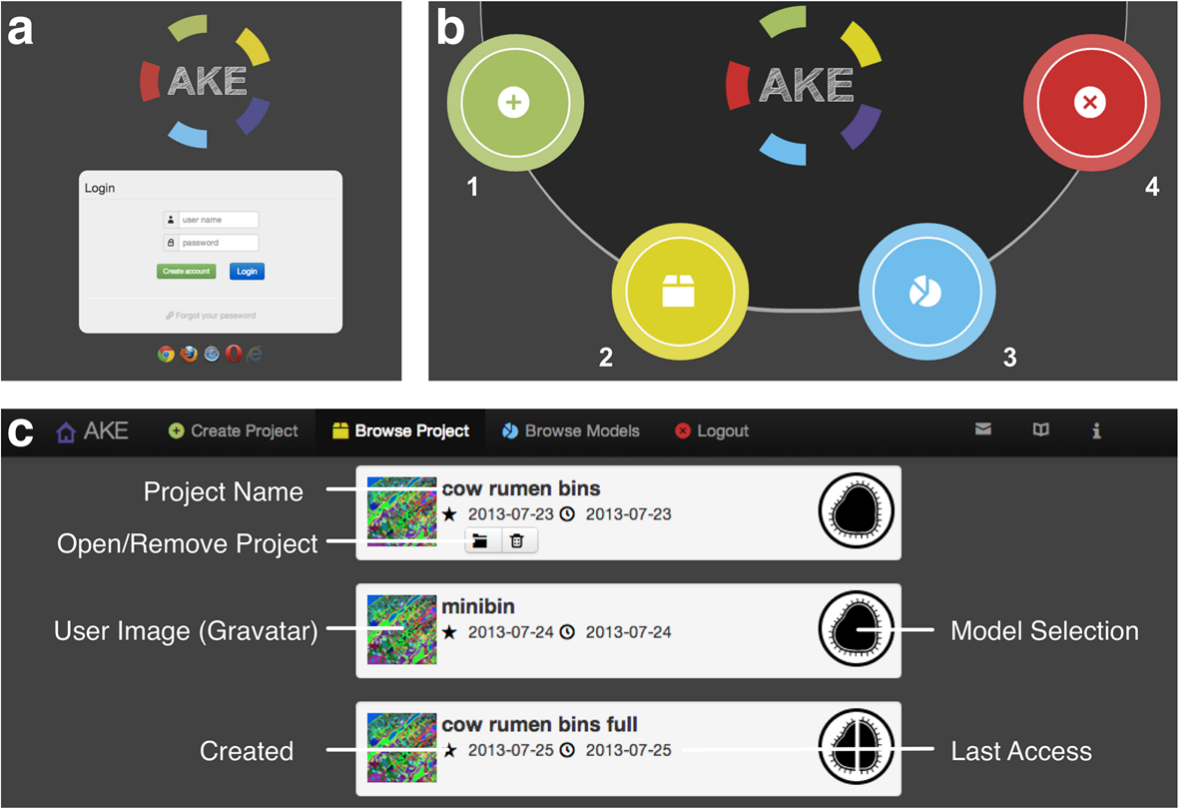
**Overview of AKE.** **a**) Login as well as registration and password retrieval can be done here. **b**) Landing Page of AKE: Here all important pages are accessible directly. 1) Create a Project 2) Browse Projects 3) Browse Models 4) Logout **c**) Project View: Basic project management features are provided.

The web-interface is accessible at www.ani.cebitec.uni-bielefeld.de/ake. The website is protected by a login screen (Figure 5a). A login with password can be chosen on this page. The browsers, which are known to work properly with AKE are indicated at the bottom of the page. After login the user is redirected to the landing page (Figure 5b) where every subpage is accessible. A basic project management – creation, removal, storage of basic information (date, last access and model selection) for the creation of the project – is supported (Figure 5c).

**Figure 6:**
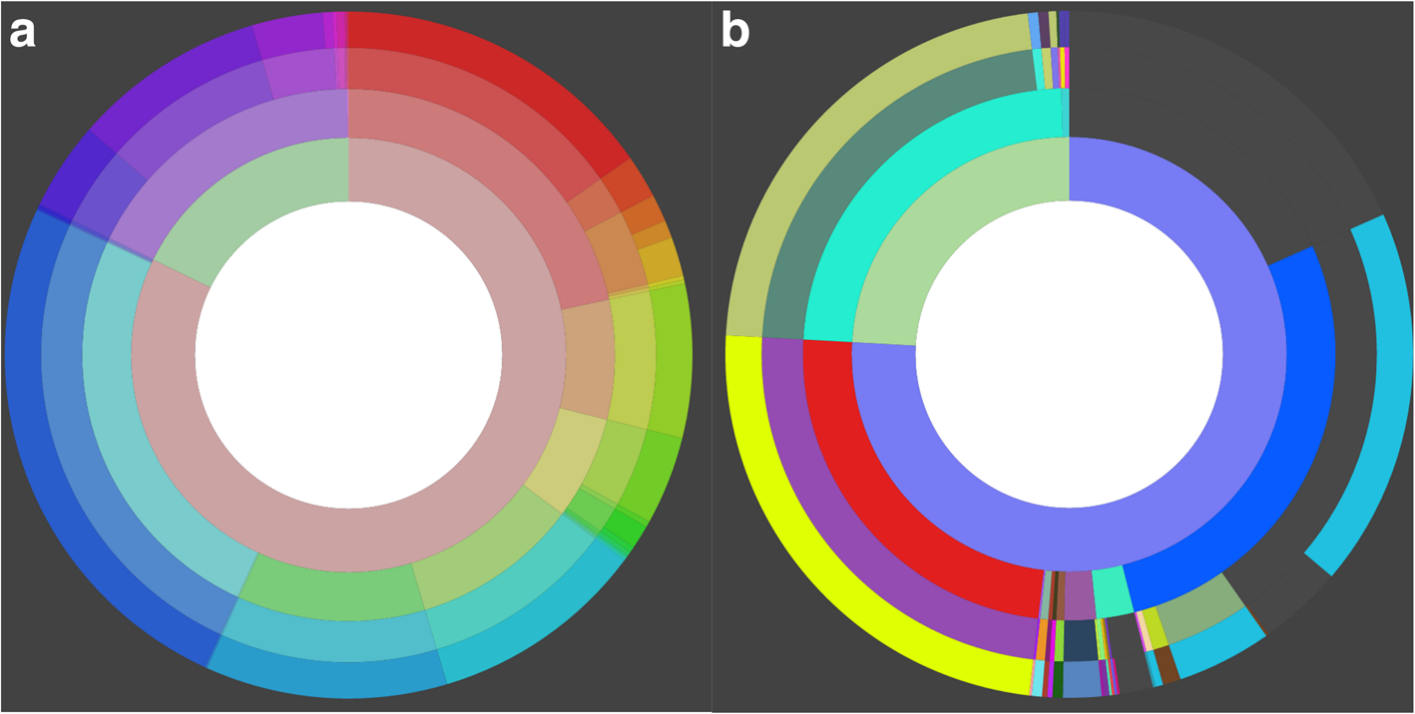
**AKE results view with coloration options.** **a**) The entities, i.e. taxonomic categories are colorized according to the position on the disc. **b**) Every entity is colorized in a predefined way.

**Figure 7:**
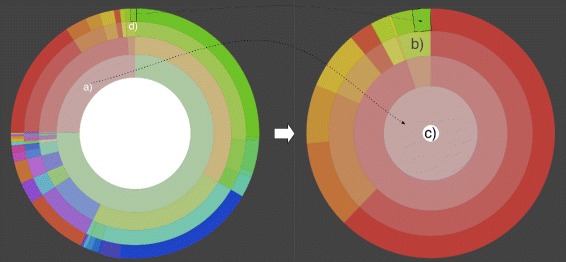
**Zooming in of AKE results view.** When clicking on one category like a) the disc is transformed. The activated category is the new root and the new whole disk is used to display only its descendant results in the taxonomic subtree. This helps in exploring smaller entities (e.g. see d)) on lower ranks. In addition, the amount of information is reduced so that the remaining one can be accessed more easily. With a click on c) one can go back to the prior view. Furthermore, one can skip taxonomic ranks on zooming in. It is not only possible to zoom in on the next rank but on arbitrary levels of the hierarchy e.g. by clicking on b).

During project creation two modes of operation can be chosen. The preview mode is for receiving a fast result for data sets smaller than 100 MB. Here the results are computed immediately. For larger files, which need more computation time, the classification mode can be used. The computation is done on a powerful machine in this mode but is not guaranteed to start immediately, so that the user will get notified by email when all results are computed. The Projects’ assignment visualizations contain a Krona [30] inspired view. For this view two different colorization options are available (Figure 6). One option colorizes every item in a specific predefined color. This is especially helpful to compare two different results as entities, because taxonomic categories are colorized consistently across results. The other option is helpful when looking at only one result and colorization is inspired by the HSV color wheel. It helps in retaining orientation when zooming in (see Figure 7). The zoom enables the user to interactively browse the classification results. By clicking on a category, it becomes the new root of the visualization. This allows the inspection of small entities and interesting subtrees. For visualization the *D*^3^ framework [31] was used. Here the so-called sunburst tree is generated with the automatic *D*^3^ partition layout. A client-server architecture is used with the back end written in Python with C-extensions. The communication is done via JSON.

## Conclusion

**Figure 8:**
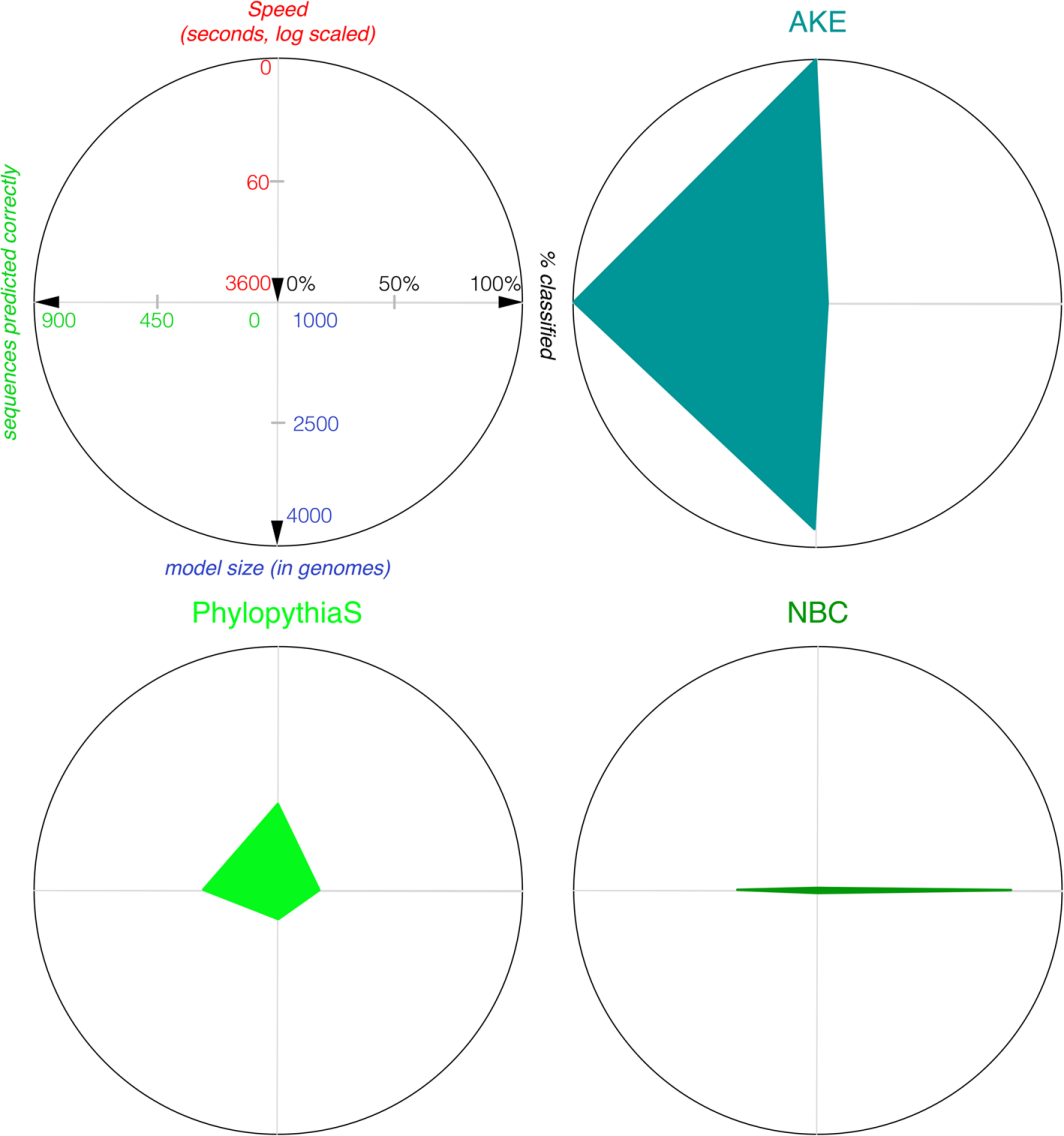
**Comparison of web-based taxonomic classifiers for AMD data set.** The data is based on [12], the respective web sites and personal measures. Note that Speed (runtime in seconds) is depicted in reverse order. Predicted correctly is the number of predictions which are also present in the reference composition. Model size is the number of genomes which are included in the model building process. The percent classified value expresses the inverse of the percentage of rejected data. (Note that differences between Figure 3 (data is cited) and this Figure (data is measured, July 2014) are most probably due to different application versions).

A comparison of web-based taxonomic classifiers is shown in Figure 8 based on the analysis of the AMD data set. AKE outperforms PhylopythiaS [11] (generic model) and NBC [12] in all measured categories and the execution time is one (PhylopythiaS) or two (NBC) orders of magnitude faster. A result with WebCarma [8], which is a homology-based classifier, has been obtained within about a week. It outperforms all composition-based methods, with 678 correct assignments, except our system AKE (902 correct assignments) on phylum level. The number of rejects of WebCarma, i.e. the assignment to an “other” unknown class, on phylum level (42%) is comparable to PhylopythiaS but it is much higher than in NBCs or AKEs results. The detailed results are given in Table 2.

**Table 2:** **Performance comparison of PhylopythiaS, NBC, WebCarma and AKE for AMD data set on phylum level**

**Algorithm**	**#Correct**	**#Wrong**	**#Unknown**	**Runtime**
	**assignments**	**assignments**	**assignments**	**(assignment)**
				**in s**
AKE	902	213	68	0.43
NBC	218	717	248	3480
PhylopythiaS	105	411	832	207
WebCarma	678	13	492	6∗10^5^

The evaluation of different web-based taxonomic classifiers shows that the runtime differs dramatically from a second (AKE), to minutes (PhylopythiaS), to an hour (NBC), to almost a week (WebCarma) due to algorithmic features and implementation details. AKE is faster compared to the other applications because it only needs to compute the euclidean distance between the descriptive model and the data that should be classified, whereas the others need to compute alignments (WebCarma) or apply decision functions (Phylopythia, NBC). Furthermore, optimized C code and multi-threading accelerates the application. The neural network used is especially suited to generate a hierarchical, compact, descriptive model, which allows fast queries using a beam search to limit the number of euclidean distance searches. Although there might be methods reported to be equally fast and more accurate, to the authors knowledge there exists no web-based solution which performs equally well, in terms of execution time *and* accuracy for generic metagenome data. Since accuracy drops down significantly for ranks lower than order we do not report these here, since our focus in development lay on acceleration and a dynamic web-based visualization system.

AKE is a fast taxonomic assignment tool for first visual inspection of whole metagenome data sets. Its web-based dynamic visualization allows fast analyses even on low performance computers without installation of software. Furthermore, the web-based approach enables a cooperative analysis of data with colleagues.

## Additional files

Additional file 1
**Detailed description of H** ^**2**^ **SOM.** PDF file giving a detailed description of the H ^2^SOM algorithm. Open with you favorite pdf reader, e.g. Adobe Reader.

Additional file 2
**Table for cross validation study.** PDF file presenting results for the cross validation study. Open with you favorite pdf reader, e.g. Adobe Reader.

Additional file 3
**List of GI numbers of sequences used for training.** Text file listing the gi numbers of the sequences used for training. Unzip and open with your favorite text editor.
