# Supplementary material for: AKE - the Accelerated k-mer Exploration web-tool for rapid taxonomic classification and visualization
Source: BMC Bioinformatics. 2014 Dec 13;15(1):384. doi: 10.1186/s12859-014-0384-0 (PMC4307196; doi:10.1186/s12859-014-0384-0)
Supplement: Additional file 1 — Detailed description of H 2 SOM. PDF file giving a detailed description of the H 2SOM algorithm. Open with you favorite pdf reader, e.g. Adobe Reader. [file 12859_2014_384_MOESM1_ESM.pdf]

H^2SOM

The Hierarchical Hyperbolic Self-Organizing Map (H^2SOM)

In this section we give a brief motivation for implementing SOMs in the hyperbolic space, as well as a brief introduction into the foundations of hyperbolic space and describe some of its features.

Foundations.
In the beginning, the hyperbolic space was just an idea which was born by the question for non-Euclidean geometry, which was basically driven by questioning the 5th axiom of Euclid. In the early 19th century, Lobachevsky, Gauss and Bolyai were the first to deny this axiom and created the theoretical notion of what is called today *hyperbolic geometry.* Later on, based on Riemannian geometry, different models have been proposed to represent the hyperbolic space in euclidean 3D with euclidean coordinates (x, y, z) computed from its original coordinates by a mapping function $x = f_x(u, v), y = f_y(u, v), z = f_z(u, v)$ from the hyperbolic space coordimates (u, v).

However, it is natural that there exists no representation of $\mathbb{H}^2$ in $\mathbb{R}^3$ which preserves all distances and angles. One of the most prominent representation models is the Poincaré Disk Model [3], which is quite similar to the Klein-Beltrami Model from 1871. The Poincaré Disk maps a point on a hyperbolic plane, given in polar coordinates (r, θ) to the open unit disk $\mathcal{D}$ [4] using the following mapping functions:

$$f_x(r, \theta) = \tanh(\frac{r}{2}) \cos(\theta)$$
$$f_y(r, \theta) = \tanh(\frac{r}{2}) \sin(\theta)$$

The distance (also called the line segment) of two points (r, θ) and $(r + \triangle r, \theta + \triangle\theta)$ is computed by

$$ds^2 = 4\frac{dr^2 + r^2 d\theta^2}{(1 - r^2)^2}. \qquad (1)$$

In the following a number of important properties of the Poincaré model are listed:

i. It maps the infinite large area of the hyperbolic plane $\mathbb{H}^2$ entirely onto the unit euclidean disc.
ii. The mapping is conformal, i. e. angles are preserved.
iii. While the angles (i. e. shape features) are preserved, the projection non-isometric and exhibits a strong fish-eye effect. The origin of the $\mathbb{H}^2$ is represented almost faithfully but with growing distance from the center, the data display gets more and more squeezed due to the tanh function.

To be able to access all details at the squeezed borders we need to implement a procedure to manipulate the Poincaré projection to allow focussing on selected areas of the $\mathbb{H}^2$.

Möbius transform.
The Möbius transform is bijective and homomorphic and it describes the group of isometries of the Poincaré disk $\mathcal{D}$ [1]. The transform maps a point z in the disc $\mathcal{D}$ to new cordinates $M(z)$ using the following formula:

$$M_{c,\phi}(z) = e^{i\varphi}\frac{z-c}{1-\bar{c}z},\ c \in \mathbb{C},\ |c| < 1. \tag{2}$$

For $c = 0$ the transform $M_{0,\phi}(z)$ just describes a rotation of $\mathbb{D}$ with angle ϕ and for $\phi = 0$ a translation is achieved, mapping c to $-c$.

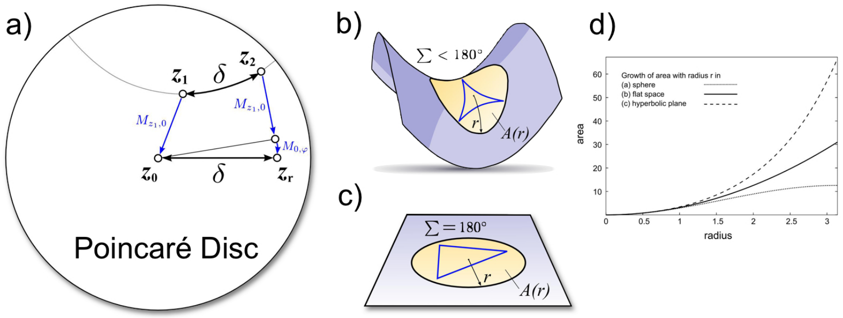

FIGURE 1. To compute distances of two points (z_1, z_2) in $\mathbb{H}^2$ one considers the circular path displayed in a). The effect on the areas with growing radius r is visualized in b) and c) and plotted in d), showing a significantly stronger increase in area size for the hyperbolic space. Please see text for details.

Properties of the hyperbolic space.
Since we choose the Poincaré disc for representing structures from the $\mathbb{H}^2$ in a euclidean plane, we need methods to compute distances of points in $\mathcal{D}$ in $\mathbb{H}^2$ as well as areas in relation to this projection as displayed in figure 1. The hyperbolic distance $\delta(z_1, z_2)$ of two points z_1 and z_2 is given by integrating the line segment (eq. (1)) along the circular path

displayed in Figure 1 a). In practice, two Möbius transforms ($M_{z_1,0}$ (translation) and $M_{0,\varphi}$ (rotation)) are applied to move z_1 to the origin z_0 and z_2 to z_m:

$$M_{0,\varphi}(M_{z_1,0(z_2)}) = e^{i\varphi}\frac{z_2 - z_1}{1 - \bar{z}_1 z_2} = z_m$$

Since $z_m = m + 0i$ is of real value it follows

$$m = \left|\frac{z_2 - z_1}{1 - \bar{z}_1 z_2}\right|$$

and integration of the line segment gives the following distance function:

$$\delta(z_1, z_2) = \int_0^m \frac{2}{1 - x^2} dx = 2\arctan(m) = 2\arctan\left(\left|\frac{z_2 - z_1}{1 - \bar{z}_1 z_2}\right|\right) \tag{3}$$

The area $A(r)$ of a hyperbolic circle with radius r (see yellow patch in Figure 1 c) is computed by

$$A(r) = \int_0^r \int_0^{2\pi} \frac{4r}{(1 - r^2)^2} dr d\theta = 4\pi \frac{r^2}{1 - r^2} = 4\pi \sinh^2\left(\frac{r}{2}\right). \tag{4}$$

From eq. 4 one can see that the circular area in hyperbolic space shows to have a feature which is of particular relevance to the self-organizing map (SOM) algorithm. For a small radius ($r < 1$) the space is almost flat and $A(r) \approx \pi r^2$. But for larger r the area grows asymptotically exponential in contrast to the quadratic growth in the flat euclidean plane (see Figure 1 c). For comparison, we display the growth rates for increasing r for a sphere, a flat space and a hyperbolic plane in Figure 1 d. So the hyperbolic plane offers more space for a SOM in a low dimensional embedding.

The Self-Organizing Map in hyperbolic space.
The classic SOM consists of a a set of formal neurons $(\mathbf{u}^{(k)}, z_k)_{k=1\ldots K}$ which are locally arranged on a regular lattice (like a kartesian grid or a hexagonal grid) with z_k representing the lattice coordinates of the kth neuron and $\mathbf{u}^{(k)}$ as the corresponding prototype vector. In the hyperbolic plane the lattice to lay out the neurons $(\mathbf{u}^{(k)}, z_k)_{k=1\ldots K}$ is constructed in the following way. First, the center node is placed (see blue node in Figure 2) in the center of $\mathcal{D}$ and a set of s nodes are placed around it. These nodes are placed as corners of $s - 1$ triangles with angle $\alpha = 360/(s - 1)$ and side length

$$l = \tanh\left(\frac{1}{2}\text{arccosh}\left(\frac{\cos(\alpha)}{1 - \cos(\alpha)}\right)\right)$$

which is practically achieved by setting the first node (green node in Figure 2a) and applying the Möbius transform $M_{0,\varphi}$ (with $\varphi = \cos(\alpha) + i\sin(\alpha)$) $(s - 1)$ times so a ring of s nodes is created. In the next step, another ring of nodes is created by expanding each perimeter node with $s - 3$ nodes, again using the Möbius transform (see Figure 2 b). This step can be repeated for each new perimeter ring of nodes until the desired number of nodes is initiated.

The final number of nodes depends on the number of neighbors s and the number of rings r and grows exponentially with r. In Figure 2c) we show a grid for $s = 7$ and $r = 2$. In 2d) we show a large HSOM with $s = 7$ and $r = 6$ to display the strong degree of squeezing the nodes in the outer perimeter at the border of the disc. Using the Möbius transform, one node can be selected as focus (red node in Figure 2d)-f) and moved to the center so the area around this node can now be inspected with a high level of detail (see Figure 2 f) while the rest of the HSOM grid is squeezed on the left side of the disc.

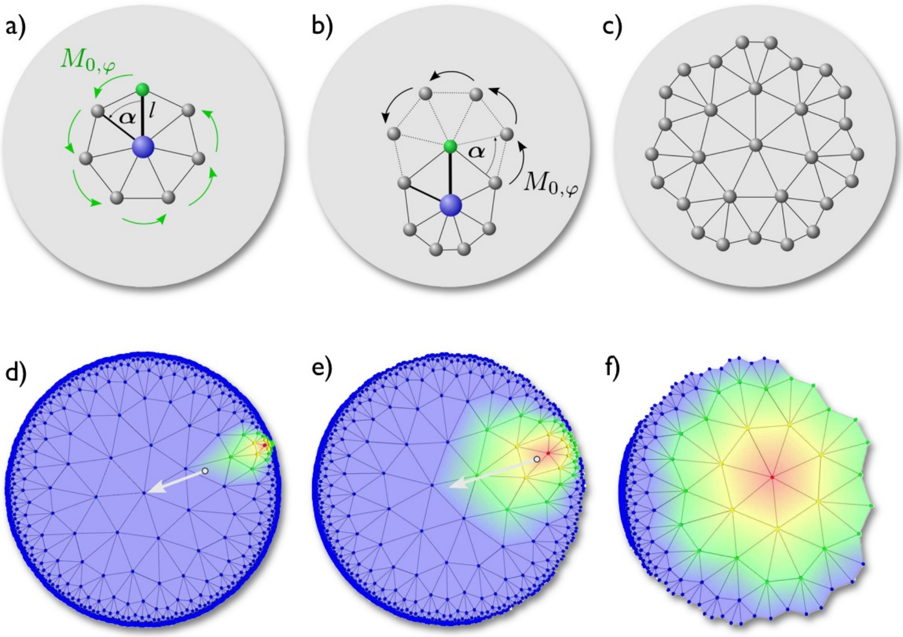

FIGURE 2. The upper row shows the construction of a HSOM grid with $s = 7$ and $r = 2$. The lower row visualizes the application of the Möbius transform to move one node in the outer periphery (red dot in d) to the center of $\mathcal{D}$ so the nodes in its vicinity can be inspected in high detail as shown in f).

The HSOM is trained basically in the same way as a "classic" SOM, but with a new neighborhood function $h(k, k^\star)$ (for two neurons k and $k^\star$) which results from exchanging the Euclidean distance in the standard Gaussian neighborhood function with the hyperbolic distance shown above (see eq. 3):

$$h(k, k^\star) = \exp\left(-\frac{\operatorname{arctan}\left(\left|\frac{z_k - z_{k^*}}{1 - \bar{z}_k z_{k^*}}\right|\right)}{\sigma^2(t)}\right). \tag{5}$$

H^{2}SOM: The hierarchical HSOM.
The grid construction concept of the HSOM results in a huge number of nodes due to the exponential growth rate. While on the one hand, a large number of nodes has the benefit of a more trustworthy embedding due to a low reconstruction error, on the other hand the training and mapping time increases painfully because of a time-consuming search for the best matching unit (BMU) in each learning step. Thus, we apply a special version of the HSOM, the hierarchical HSOM (H^{2}SOM) which uses a beam search strategy when searching for the best match unit. This means, that for each training step the BMU is searched iteratively starting with the inner 1st ring. When the BMU in the 1st ring is found, only the successors (i. e. child) in the 2nd ring are analyzed regarding the BMU criterion, and so forth 3. In [2] it was shown, that these H^{2}SOM have the same potential to perform complex unsupervised learning and projection tasks compared to the original SOM but have log scale training time.

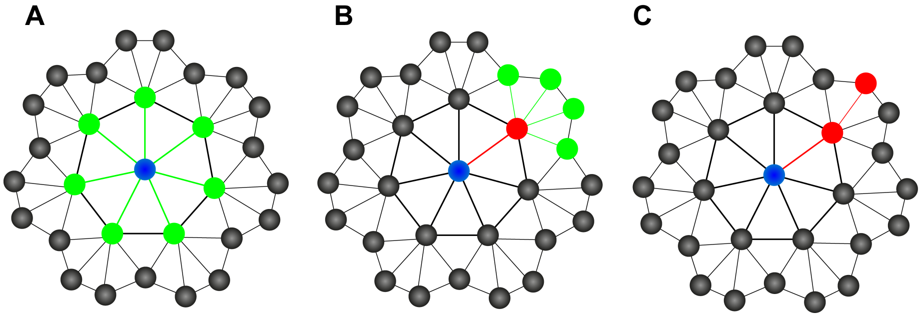

FIGURE 3. Beam Search with width=1. With beam search only a subset of all nodes is visited during BMU search. The red colored nodes are the BMUs of the current ring. The BMU for the next ring is searched only in the green colored nodes.

References

[1] J. Anderson. *Hyperbolic Geometry.* Springer Verlag, New York, 2001.
[2] J. Ontrup and H. Ritter. Large-scale data exploration with the hierarchically growing hyperbolic SOM. *Neural Networks*, 19:751–761, 2006.
[3] H. Poincaré. Sur les applications de la gométrie non euclidienne la thorie des formes quadratiques. *Compte Rendu de lassociation Francaise pour lAvancement des Sciences*, 10:132138, 1881.
[4] A. Ramsay and R. Richtmyer. *Introduction to Hyperbolic Geometry.* Springer Verlag, New York, 1995.
