# Supplementary material for: AKE - the Accelerated k-mer Exploration web-tool for rapid taxonomic classification and visualization
Source: BMC Bioinformatics. 2014 Dec 13;15(1):384. doi: 10.1186/s12859-014-0384-0 (PMC4307196; doi:10.1186/s12859-014-0384-0)
Supplement: Additional file 2 — Table for cross validation study. PDF file presenting results for the cross validation study. Open with you favorite pdf reader, e.g. Adobe Reader. [file 12859_2014_384_MOESM2_ESM.pdf]

| Architecture | | | | | | | F1 Score | | | | | | | |
|---|---|---|---|---|---|---|---|---|---|---|---|---|---|---|
| Rings | Spread | Alpha | Beta | k-mer size | labeling | classification | Domain | Rejects | Phylum | Rejects | Class | Rejects | Order | Rejects |
| 2 | 8 | 0.8 | - | 4 | purity | nn | 0.94 | 0 | 0.9 | 597603 | 0.92 | 600533 | 0.8 | 601226 |
| 3 | 8 | 0.8 | - | 4 | purity | nn | 0.94 | 0 | 0.9 | 548698 | 0.88 | 580651 | 0.9 | 586970 |
| 4 | 8 | 0.8 | - | 4 | purity | nn | 0.95 | 0 | 0.92 | 476725 | 0.89 | 534357 | 0.9 | 552526 |
| 5 | 8 | 0.8 | - | 3 | purity | nn | 0.95 | 0 | 0.91 | 439761 | 0.88 | 495535 | 0.86 | 516786 |
| 5 | 8 | 0.8 | - | 4 | purity | nn | 0.96 | 0 | 0.9 | 382273 | 0.89 | 435024 | 0.87 | 142649 |
| 5 | 8 | - | - | 4 | majority | nn | 0.97 | 0 | 0.72 | 0 | 0.64 | 0 | 0.57 | 0 |
| 5 | 8 | 0.8 | 0.075 | 4 | purity | thresh | 0.97 | 26501 | 0.92 | 393527 | 0.89 | 445645 | 0.88 | 470805 |
| 5 | 8 | 0.8 | - | 4 | purity | nbrs | 0.96 | 0 | 0.91 | 391079 | 0.88 | 450484 | 0.86 | 474481 |
| 5 | 8 | 0.8 | - | 5 | purity | nn | 0.96 | 0 | 0.92 | 434610 | 0.91 | 492016 | 0.88 | 517578 |
| 5 | 8 | 0.9 | - | 4 | purity | nn | 0.96 | 0 | 0.9 | 448278 | 0.88 | 481285 | 0.87 | 495519 |
| 5 | 8 | 0.99 | - | 4 | purity | nn | 0.96 | 0 | 0.93 | 522000 | 0.93 | 542684 | 0.92 | 556942 |
| 6 | 8 | 0.8 | - | 4 | purity | nn | 0.97 | 0 | 0.9 | 395223 | 0.88 | 451020 | 0.86 | 126644 |
